# Supplementary material for: Propolis, Bee Honey, and Their Components Protect against Coronavirus Disease 2019 (COVID-19): A Review of In Silico, In Vitro, and Clinical Studies
Source: Molecules. 2021 Feb 25;26(5):1232. doi: 10.3390/molecules26051232 (PMC7956496; doi:10.3390/molecules26051232)
Supplement: Supplementary file 1 [file molecules-26-01232-s001.zip › Suppl/Supplementary file 1.pdf]

Further details of studies simulating the binding of bee-related compounds to SARS-CoV-2-related proteins

Our literature search resulted in seven studies of molecular modeling. Most studies retrieved the two-dimensional (2D) or 3D structure of evaluated bee-derived compounds as SD files from online databases such as ZINC database [30] (http://zinc15.docking.org/) or PubChem database (https://www.ebi.ac.uk/chembl/) [2,5,32,55]. The 2D structures were edited in software supplemented with data on ionization, variation, stereochemical correction, and energy minimization to yield a 3D structure [5,32,55]. Ligands were further geometrically optimized for docking by various software e.g., MarvinSketch, Autodock Tools 1.5.6, Autodock Vina [50], MOPAC2016 software [30], PyMOL software [2], LigPrep module of Schrodinger Maestro v10.1 [5,32], ChemDraw 3D ultra 9.0, Molsoft Internal Coordinate Mechanics (ICM) 3.4-8C [58], and Hyperchem software [55].

Five studies used agents with known inhibiting activities of intended proteins of SARS-CoV-2 or host ACE-II receptor or serein proteases as positive controls: 1) Camostat mesylate for TMPRSS2 inhibition [5], 2) (*S*, *S*) -2- [1-Carboxy-2- [3- (3,5-Dichloro-Benzyl) -3h-Imidazole-4-Yl] -Ethylamino]xx-4-Methyl-Pentanoic Acid (known as MLN-4760), tert-butyl(1-((*S*)-1-(((*S*)-4-(benzylamino)-3,4-dioxo-1-((*S*)-2-oxopyrrolidin-3-yl)butan-2-yl)-amino)-3-cyclopropyl-1-oxopropan-2-yl)-2-oxo-1,2-dihydro-pyridin-3-yl) carbamate (known as 13b), 14b [50], avigan, hydroxychloroquine, and remdesivir for ACE-II binding [55], 3) theaflavin for SARS-CoV-2 RdRp binding, and 4) ligand X77, N3 (the ligand of 6lu7—the active site of 3CL-pro), avigan, hydroxychloroquine, and remdesivir for 3CLpro inhibition [30]. A single study compared the affinity of artepillin C to PP2A B56 with that of ^{293}LDPLSE^{298}, which is located in S1 subdomain of S protein [2].
