# Supplementary material for: Propolis, Bee Honey, and Their Components Protect against Coronavirus Disease 2019 (COVID-19): A Review of In Silico, In Vitro, and Clinical Studies
Source: Molecules. 2021 Feb 25;26(5):1232. doi: 10.3390/molecules26051232 (PMC7956496; doi:10.3390/molecules26051232)
Supplement: Supplementary file 1 [file molecules-26-01232-s001.zip › Suppl/Supplementary Table 1.pdf]

Supplementary Table 1. Interactions of flavonoids from propolis and bee honey with host-cell receptors proteins of SARS-CoV-2

Proteins (PDB ID)	Compounds	Binding energy (kcal/mol)	Main interactions	References
ACE-II (1R4L)	MLN-4760*▲	-7.28	Tyr127, Arg273, Phe274, Trp271, Arg273, Phe274, His345, Pro346, Cys361, Thr371, His374, Glu375, His378, Glu406, Phe504, His505, Tyr515, Arg518	[1]
ACE-II (1R4L)	Rutin	-8.98	HB (Asn149, His345, Asp269, Glu375, Glu406, Thr371, Tyr127, Asp368), PC (Arg273), PPT (His374), Al (Cys344), PAl (Tyr 127)<br>Tyr127, Ser128, Leu144, Glu145, Asn149, Asp269, Met270, Trp271, Arg273, Phe274, Val343, Cys344, His345, Pro346, Met360, Cys361, Lys363, Asp367, Asp368, Thr371, His374, Glu375, Glu406, Phe504, Arg518 ▲	[1]
ACE-II (1R4L)	CAPE▲	-7.76	Tyr127, Ser128, Leu144, Glu145, Asn149, Trp271, Val343, Cys344, His345, Pro346, Met360, Cys361, Thr362, Lys363, Asp368, Phe504	[1]
ACE-II (1R4L)	Myricetin▲	-7.70	Arg273, Phe274, His345, Pro346, Thr347, Ala348, Thr371, His374, Glu375, His378, Glu406, Phe504, His505, Tyr515, Arg518	[1]
ACE-II (1R4L)	Quercetin▲	-7.62	Arg273, Phe274, His345, Pro346, Thr347, Ala348, Thr371, Glu375, His374, His378, Glu406, Phe504, His505, Tyr515, Arg518	[1]

Proteins (PDB ID)	Compounds	Binding energy (kcal/mol)	Main interactions	References
ACE-II (1R4L)	Pinocembrin▲	-7.46	Tyr127, Ser128, Leu144, Glu145, Asn149, Trp271, Val343, Cys344, His345, Pro346, Met360, Cys361, Thr362, Lys363, Asp368	[1]
ACE-II (1R4L)	Hesperetin▲	-7.40	Arg273, His345, Pro346, Thr347, Ala348, Trp349, Thr371, His374, Glu375, His378, Glu406, Phe504, His505, Tyr510, Tyr515, Arg518	[1]
ACE-II (1R4L)	Galangin▲	-7.18	Arg273, Phe274, His345, Pro346, Thr347, Ala348, Thr371, His374, Glu375, His378, Glu406, Phe504, His505, Tyr515, Arg518	[1]
ACE-II (1R4L)	Chrysin▲	-7.08	Tyr127, Ser128, Glu145, Asn149, Cys344, His345, Pro346, Met360, Cys361, Thr362, Lys363, Asp368, Phe504	[1]
ACE-II (1R4L)	Luteolin▲	-6.93	Arg273, Phe274, His345, Pro346, Thr347, Ala348, Trp349, His374, Glu375, His378, Asp382, Glu402, Phe504, His505, Tyr510, Tyr515, Arg518	[1]
ACE-II (1R4L)	Caffeic acid▲	-5.53	Arg273, His345, Pro346, Thr347, Ala348, His374, Glu375, His378, Phe504, His505, Tyr515	[1]
TMPRSS2 (O15393)	Camostat mesylate *	-5.90	HB (Gly464), PP (Val275, Gln276, Val278, Val 280, His296, Cys297, Leu302, Asp435, Ser436, Cys437, Gln438, Gly439,	[2]

Proteins (PDB ID)	Compounds	Binding energy (kcal/mol)	Main interactions	References
			Ser441, Thr459, Trp461, Gly462, Cys465, Ala466, Gly472, Val473)	
TMPRSS2 (O15393)	CAPE	-6.20	HB (Gly464, Ser436), PP (Cys281, Val280, His296, Cys297, Glu299, Leu302, Asp435, Cys437, Gln438, Gly439, Asp440, Ser441, Thr459, Ser460, Trp461, Gly462, Cys465	[2]
S1 subunit (6m0j)	**Dexamethasone***	−7.9		[3]
S1 subunit (6m0j)	**Naringin**	−9.8	HB (Asp367, Thr371, Lys441, Ser409, Glu406) hydrophobic (Asn290, Ile291, His374, Leu370, Leu410, Ala413, Pro415, Phe438, Gln442)	[3]
S1 subunit (6m0j)	**Rutin**	−9.2	NR	
S1 subunit (6m0j)	**Galangin**	−8.2	NR	
S1 subunit (6m0j)	**Quercetin**	−8.2	NR	
S1 subunit (6m0j)	**Chrysin**	−8.1	NR	
S1 subunit (6m0j)	**Luteolin**	−8.0	NR	
S1 subunit (7BZ5)	Remdesivir*	−165.9◎	3HB (Arg509, Asn343, Ala344)	[4]
S1 subunit (7BZ5)	Avigan*	−46.3◎	3HB (Arg509)	[4]
S1 subunit (7BZ5)	Hydroxychloroquine*	−79.8◎	2HB (Arg509)	[4]

Proteins (PDB ID)	Compounds	Binding energy (kcal/mol)	Main interactions	References
S1 subunit (7BZ5)	Rutin	−94.3◎	1HB (Glu484)	[4]
S1 subunit (7BZ5)	CAPE	−77.8◎	2HB (Asn437, Ala372)	[4]
S1 subunit (7BZ5)	Pinobanksin	−77.4◎	3HB (Trp436, Phe342)	[4]
S1 subunit (7BZ5)	Quercetin	−67.8◎	2HB (Gln474)	[4]
S1 subunit (7BZ5)	Chrysin	−66.2◎	1HB (Asn343)	[4]
S1 subunit (7BZ5)	Kaempferol	−62.3◎	2HB (Trp436)	[4]
S1 subunit (7BZ5)	Pinocembrin	−60.5◎	1HB (Gly482)	[4]
S1 subunit (7BZ5)	Galangin	−59.5◎	1HB (Gly482)	[4]
S1 subunit (7BZ5)	P-coumaric acid	−56.5◎	3HB (Arg509, Ser373)	[4]
PP2A-B56 (5SWF-A)	^{293}LDPLSE^{298}◆	-4.9	Leu296 formed HB with the hydrophobic pocket, and Glu298 formed ionic bonds with amino acid residues in the positively-charged region in PP2A B56. ○	[5]
PP2A-B56 (5SWF-A)	Artepillin C	-6.1	The two prenyl groups of artepillin C were docked into the same two pockets as in ^{293}LDPLSE^{298}; however, only through HB. ○	{Maaroufi, 2020 #1892
Mpro (6YNQ)	Remdesivir*	– 8.6	HB (Glu A: 166, His A: 41, Thr A: 25) PA (Pro A: 168)	[6]

Proteins (PDB ID)	Compounds	Binding energy (kcal/mol)	Main interactions	References
Mpro (6YNQ)	Rutin	− 8.7	HB (CYS A: 145, HIS A: 163, SER A: 46, THR A: 45, HIS A: 41, GLU A: 166, And GLN A: 189) Van der Waals (Asn A: 142.) PA (Met A: 49)	[6]
Mpro (6Y2F)	13b*	−8.2	HB (His41, Gly143, Ser144, Cys145, Glu166) HDPC (Thr25, Cys44, Met49, Leu141, Asn142, His163, His164, Met165, Leu167, Asp187, Gln189)	[7]
Mpro (6Y2F)	14b*	−7.2	HB (Phe140, Glu166) HDPC (His41, Met49, Leu141, Asn142, His163, His164, Met165, Leu167, Pro168, Asp187, Arg188, Gln189)	[7]
Mpro (6Y2F)	Broussoflavonol F	−7.8	HB (Gly143, Ser144, Cys145) HDPC (Thr26, His41, Met49, Leu141, Asn142, His163, His164, Met165, Glu166, Arg188, Gln189)	[7]
Mpro (6Y2F)	Glyasperin A	−7.8	HB (Thr25, His164, Arg188) HDPC (His41, Cys145, Met165, Glu166, Val186, Asp187, Gln189, Thr190, Gln192)	[7]
Mpro (6Y2F)	Sulabiroins A	−7.6	HB (Glu166) HDPC (His41, Asn142, His164, Met165, Asp187, Arg188, Gln189)	[7]
RdRp(7M71)	Theaflavin*	-11.2	HB (Ala550, Trp617, Aap623, Asp760, Glu811), PA (Asp760, Asp761), PAl (Ala550, Cys622)	[8]
RdRp(7M71)	Rutin	-11.7	HB (Tyr619, Lys621, Asp760, Glu811), PA (Glu811)	[8]

Proteins (PDB ID)	Compounds	Binding energy (kcal/mol)	Main interactions	References
RdRp(7M71)	Quercetin-7-O-glucuronide	-10.5	HB (Arg553, Ser549, Tyr619, Trp800, Glu811, Cys813), PC (ARG555, LYS551)	[8]
RdRp(7M71)	Quercetin-3'-O-glucuronide	-10.5	HB (Cys622, Asp623, Asp760, Glu811, Cys813, Ser814), PA (Asp618, Asp761)	[8]
RdRp(7M71)	Quercetin-3-O-glucuronide	-10.2	HB (Thr556, Tyr619, Lys621, Thr687, Asn691, Ser759, Asp760), PC (Arg555, Arg553), PA (Asp623), PAl (Lys621)	[8]
RdRp(7M71)	Quercetin-7-O-sulfate	-9.4	HB (Lys545, Lys621, Arg624), PC (Arg555, Arg553), PA (Asp623), PAl (Arg624)	[8]
RdRp(7M71)	Quercetin-3-O-sulfate	-8.8	HB (Asp452, Arg553, Thr556, Tyr619), PC (Arg553), PA (Asp623)	[8]
RdRp(7M71)	Quercetin-3'-O-sulfate	-8.8	HB (Ser814), PA (Asp760, Asp761)	[8]
RdRp(7M71)	Quercetin	-8.2	HB (Asp760, Trp800), PA (Asp761)	[8]
RdRp(7M71)	Nicotiflorin	-11.3	HB (Tyr619, Lus621, Asp760), PA (Glu811)	[8]
RdRp(7M71)	Kaempferol-4'-O-glucuronide	-10.2	HB (Asp618, Lys621, Asp623, Asp761), PA (Glu811)	[8]
RdRp(7M71)	Kaempferol-3-O-glucuronide	-10.1	HB (Tyr619, Asp760, Asp761, Glu811, Cys813)	[8]

Proteins (PDB ID)	Compounds	Binding energy (kcal/mol)	Main interactions	References
RdRp(7M71)	Kaempferol-7-O-glucuronide	-10.0	HB (Asp760, Trp800), PA (Glu811), PAl (Lys798)	[8]
RdRp(7M71)	Kaempferol-7-O-sulfate	-9.3	HB (Lys545, Lys621, Arg624), PC (Arg553, Arg555), PA (Asp623), PAl (Arg624)	[8]
RdRp(7M71)	Kaempferol-4'-O-sulfate	-9.2	HB (Arg553, Lys621, Thr687, Asn691), PC (Arg553), PA (Asp623), PS (Cys622), PAl (Lys621)	[8]
RdRp(7M71)	Kaempferol-3-O-sulfate	-8.7	HB (Arg553, Arg555, Thr556, Tyr619, Lys621, Arg624), PC (Arg553), PA (Asp623), PAl (Lys621)	[8]
RdRp(7M71)	Kaempferol	-7.7	HB (Tyr619, Ser814), PA (Asp760, Asp761)	[8]
3CLpro(6W63)	x77*	-12.4	HB (Gly143, Glu166), PS (Met49), PAl (Leu27, His41, Pro168), PAm (Leu141)	[8]
3CLpro(6W63)	Rutin	-10.3	HB (Asn142, Asp187), PS (Cys145, Met165), PP (His41), PC (His41), PAl (Cys44, Met49, Cys145)	[8]
3CLpro(6W63)	Quercetin-7-O-glucuronide	-10.9	HB (Thr26, Glu166, Gln189), PP (His41), PC (His41), PAl (Met49)	[8]
3CLpro(6W63)	Quercetin-3'-O-glucuronide	-10.2	HB (Gly143, His164), PAl (Met49)	[8]

Proteins (PDB ID)	Compounds	Binding energy (kcal/mol)	Main interactions	References
3CLpro(6W63)	Quercetin-3-O-glucuronide	-10.7	HB (Thr25, Cys44, Asn142, Ser144, His163, Met165, Glu166, Arg188), PAl (Met49, Met165)	[8]
3CLpro(6W63)	Quercetin-7-O-sulfate	-9.5	HB (Thr25, Ser144, Glu166), PS (Cys145), PC (His41), PAl (Met49, Met165)	[8]
3CLpro(6W63)	Quercetin-3-O-sulfate	-9.5	HB (Asn142, Thr190), PS (Met165), Psi (Gln189), PAl (Met165)	[8]
3CLpro(6W63)	Quercetin-3'-O-sulfate	-9.2	HB (Met49, Gly143, Asp187), Psi (Thr25), PP (His41), PC (His41), PAl (Cys44, Met49)	[8]
3CLpro(6W63)	Quercetin	-8.3	HB (Glu166), PS (Met49), PC (His41), PAl (Met165)	[8]
3CLpro(6W63)	Nicotiflorin	-11.2	HB (Asn142, Asp187), PP (His41), PS (Cys145), PC (His41), PAl (Cys44, Met49, Cys145)	[8]
3CLpro(6W63)	Kaempferol-7-O-glucuronide	-10.9	HB (Thr24, Asp187), PP(His41), PS(Cys145), PC (His41), PAl (Cys44, Met49, Cys145)	[8]
3CLpro(6W63)	Kaempferol-4'-O-glucuronide	-9.7	HB (Thr24, Cys44), PS(Met49), PP(His41), PAl (Cys44, Met49)	[8]
3CLpro(6W63)	Kaempferol-3-O-glucuronide	-9.1	HB (Asn142, Glu166), PP (His41), PC(His41), PAl (Cys44, Met49)	[8]
3CLpro(6W63)	Kaempferol-7-O-sulfate	-9.1	HB (Gly143, Glu166) ,PS (Cys145), PP (His41), PC (His41), PAl (Cys44, Met49, Cys145)	[8]

Proteins (PDB ID)	Compounds	Binding energy (kcal/mol)	Main interactions	References
3CLpro(6W63)	Kaempferol-4'-O-sulfate	-9.0	HB (Leu141, His163), PS (Cys145), PP (His41)	[8]
3CLpro(6W63)	Kaempferol-3-O-sulfate	-9.4	HB (Met49, His41, Gly143, Glu166), PS (Cys44, Met49, Cys145), PP (His41), PC (His41), PAl (Cys145)	[8]
3CLpro(6W63)	Kaempferol	-8.1	HB (Met49, Gly143, Asp187), PS (Cys44, Cys145), PP (His41), PC (His41), PAl (Cys145)	[8]
3CLpro(6LU7)	N3*	−133.6◎	5HB (Gly71, Lys97, Gly11, Glu14)	[4]
3CLpro(6LU7)	Remdesivir*	−136.4◎	10HB (Gln110, Thr111, Thr292, Phe294, Asp153, Ser158)	[4]
3CLpro(6LU7)	Avigan*	−33.3◎	6HB (Gln110, Thr111, Thr292, Asn151, Asp295)	[4]
3CLpro(6LU7)	Hydroxychloroquine*	−65.9◎	3HB (Gln110, Thr111, Thr292)	[4]
3CLpro(6LU7)	Rutin	−92.8◎	18HB (Lys102, Gln110, Asn151, Ser158, Thr111, Ile152, Asp153)	[4]
3CLpro(6LU7)	CAPE	−67.8◎	5HB (Lys102, Asp153)	[4]
3CLpro(6LU7)	Quercetin	−57.5◎	10HB (Lys102, Gln110, Thr111, Asp153, Asp295)	[4]
3CLpro(6LU7)	Kaempferol	−56.3◎	7HB (Lys5, Lys137, Tyr239, Glu288, Glu290)	[4]
3CLpro(6LU7)	Pinocembrin	−56.2◎	3HB (Gly71, N119, Ser121)	[4]

Proteins (PDB ID)	Compounds	Binding energy (kcal/mol)	Main interactions	References
3CLpro(6LU7)	Pinobanksin	−54.1◎	5HB (Gln110, Thr111, Ser158)	[4]
3CLpro(6LU7)	Galangin	−53.2◎	4HB (Gly71, Glu14, Gly15, Ser121)	[4]
3CLpro(6LU7)	Chrysin	−52.9◎	8HB (Lys102, Gln110, Thr111, S158, Asp295)	[4]
3CLpro(6LU7)	P-Coumaric acid	−45.5◎	5HB (Thr111, Thr292, Ser158)	[4]
3CLpro(6LU7)	Benzoic acid	−35.4◎	4HB (Gln110, Thr111)	[4]
Mpro (5R7Y)	3-phenyllactic acid	-5.867	HB (GLN-189), PP(HIE-41)	[8]
Mpro (5R7Y)	Caffeic acid	-4.387	HB (GLN-189, HIE-164)	[8]
Mpro (5R7Y)	CAPE	-6.386	HB (THR-24, THR-26), PP(HIE-41)	[8]
Mpro (5R7Y)	Chrysin	-6.097	HB (SER-46, THR-24, THR-26), PP(HIE-41)	[8]
Mpro (5R7Y)	Galangin	-6.307	HB (SER-46, THR-24) , PP(HIE-41)	[8]

* Reference compound; ◎ affinity of binding is expressed as Molsoft Internal Coordinate Mechanics (ICM) score; ▲ reports on the nature of binding interactions are missing; ◆ a motif in S1 subdomain of spike protein; ○ the study did not state amino acid residues in PP2A B56 that were involved in the interactions with ^{293}LDPLSE^{298} and artepillin C. **Abbreviations:** ACE-II: angiotensin-converting enzyme-related carboxypeptidase; PP2A-B56: B56 regulatory unit of phosphatase 2A; TMPRSS2: transmembrane protease serine 2; 3CLpro/Mpro: chymotrypsin-like protease/main protease; RdRp: RNA-dependent RNA polymerase; CAPE: caffeic acid phenethyl ester; MLN-4760: ((S,S)-2-1-Carboxy-2-[3-(3,5-Dıchloro-Benzyl)-3h-Imıdazol-4-Yl]-Ethylamıno-4-Methyl-Pentanoic Acid); HB: hydrogen bond; PS: π-sulfur; PA: π-anion; PC: π-cation; PP: π-π; AL: alkyl; PAl: π-alkyl; PPT: π-π T shape; Psi: π-sigma; PAm: π-amide; HDPC: hydrophobic interactions that were not specified.

References

1. Güler, H.I.; Tatar, G.; Yildiz, O.; Belduz, A.O.; Kolayli, S. Investigation of potential inhibitor properties of ethanolic propolis extracts against ACE-II receptors for COVID-19 treatment by Molecular Docking Study. *ScienceOpen* **2020**, doi:10.14293/S2199-1006.1.SOR-.PP5BWN4.v1.
2. Kumar, V.; Dhanjal, J.K.; Bhargava, P.; Kaul, A.; Wang, J.; Zhang, H.; Kaul, S.C.; Wadhwa, R.; Sundar, D. Withanone and withaferin-A are predicted to interact with transmembrane protease serine 2 (TMPRSS2) and block entry of SARS-CoV-2 into cells. *J Biomol Struct Dyn* **2020**, 1-27, doi:10.1080/07391102.2020.1775704.
3. Jain, A.S.; Sushma, P.; Dharmashekar, C.; Beelagi, M.S.; Prasad, S.K.; Shivamallu, C.; Prasad, A.; Syed, A.; Marraiki, N.; Prasad, K.S. In silico evaluation of flavonoids as effective antiviral agents on the spike glycoprotein of SARS-CoV-2. *Saudi J Biol Sci* **2021**, *28*, 1040-1051, doi:https://doi.org/10.1016/j.sjbs.2020.11.049.
4. Refaat, H.; Mady, F.M.; Sarhan, H.A.; Rateb, H.S.; Alaaeldin, E. Optimization and evaluation of propolis liposomes as a promising therapeutic approach for COVID-19. *Int J Pharm* **2020**, 120028-120028, doi:10.1016/j.ijpharm.2020.120028.
5. Maaroufi, H. The Spike Protein S1 Subunit of SARS-CoV-2 Contains an LxxIxE-like Motif that is Known to Recruit the Host PP2A-B56 Phosphatase. *bioRxiv* **2020**, 2020.2004.2001.020941, doi:10.1101/2020.04.01.020941.
6. Arora, S.; Lohiya, G.; Moharir, K.; Shah, S.; Yende, S. Identification of Potential Flavonoid Inhibitors of the SARS-CoV-2 Main Protease 6YNQ: A Molecular Docking Study. *DCM* **2020**, *3*, 239-248, doi:https://doi.org/10.1016/j.dcmed.2020.12.003.
7. Sahlan, M.; Irdiani, R.; Flamandita, D.; Aditama, R.; Alfarraj, S.; Ansari, M.J.; Khayrani, A.C.; Pratami, D.K.; Lischer, K. Molecular interaction analysis of Sulawesi propolis compounds with SARS-CoV-2 main protease as preliminary study for COVID-19 drug discovery. *J. King Saud Univ. Sci.* **2021**, *33*, 101234, doi:https://doi.org/10.1016/j.jksus.2020.101234.
8. Silva, F.M.A.d.; Silva, K.P.A.d.; Oliveira, L.P.M.d.; Costa, E.V.; Koolen, H.H.F.; Pinheiro, M.L.B.; Queiroz, A.; Souza, L.d.; Souza, A.D.L.d. Flavonoid glycosides and their putative human metabolites as potential inhibitors of the SARS-CoV-2 main protease (Mpro) and RNA-dependent RNA polymerase (RdRp). *Mem Inst Oswaldo Cruz E-pub* **2020**, *115:e200207*, doi:10.1590/0074-02760200207.
